# Retracted: Slope Stability Analysis Using Limit Equilibrium Method in Nonlinear Criterion

**DOI:** 10.1155/2015/419636

**Published:** 2015-01-20

**Authors:** The Scientific World Journal

The paper titled “Slope Stability Analysis Using Limit Equilibrium Method in Nonlinear Criterion” [1], published in The Scientific World Journal, has been retracted upon the authors' request as it was found to include erroneous data. Its findings and conclusion cannot be relied on.
